# Infectious Bronchitis Hatchery Vaccination: Comparison between Traditional Spray Administration and a Newly Developed Gel Delivery System in Field Conditions

**DOI:** 10.3390/vetsci8080145

**Published:** 2021-07-29

**Authors:** Matteo Legnardi, Henrik Baranyay, Csanád Simon, János Molnár, Tiede Bijlsma, Mattia Cecchinato, András Gáspárdy, András Bersényi, Claudia Maria Tucciarone, Giovanni Franzo, László Kőrösi

**Affiliations:** 1 Department of Animal Medicine, Production and Health (MAPS), University of Padua, 35020 Legnaro (PD), Italy; matteo.legnardi@phd.unipd.it (M.L.); mattia.cecchinato@unipd.it (M.C.); claudiamaria.tucciarone@unipd.it (C.M.T.); giovanni.franzo@unipd.it (G.F.); 2 Phlatus ZRT, Kisperkáta telep 1., 2431 Perkáta, Hungary; henrik@phlatus.hu (H.B.); simon@phlatus.hu (C.S.); 3 Laboratory of Plastic and Rubber Technology, Department of Physical Chemistry and Materials Science, Budapest University of Technology and Economics, Műegyetem rkp. 3.H.ép, H-1111 Budapest, Hungary; molnar.janos@mail.bme.hu; 4 BioChek B.V. Fokkerstraat 14, 2811 ER Reeuwijk, The Netherlands; tiedebijlsma@biochek.com; 5 Department of Animal Breeding and Genetics, University of Veterinary Medicine Budapest, István utca 2., H-1078 Budapest, Hungary; gaspardy.andras@univet.hu (A.G.); bersenyi.andras@univet.hu (A.B.); 6 AgriAL Bt, Béri Balogh Ádám u 42, 2100 Gödöllő, Hungary

**Keywords:** Infectious bronchitis virus, gel vaccination, spray vaccination, broiler, hatchery

## Abstract

The control of infectious bronchitis (IB) is essential in intensive broiler production and is pursued through strict biosecurity and mass vaccination. Despite effective and routinely adopted, hatchery spray vaccination has been hypothesized to affect chicks’ body temperature and wellbeing. Recently, gel administration has been proposed as an alternative and proved feasible in experimental settings. In this study, IBV spray and gel vaccination methods were compared in field conditions. One hundred birds from the same hatch were enrolled in the study and vaccinated, half by spray and half by gel, with 793B and Mass vaccines. After vaccination, rectal temperature was measured and vaccine intake assessed. The two groups were housed for 35 days in separate pens and swabs and blood samples were collected at multiple time points for genotype-specific molecular analyses and serology, respectively. The temperature was significantly lower in spray-vaccinated chicks 10 min and an hour after administration. A similar trend in 793B titres was observed in both groups, while the Mass vaccine was detected later but persisted longer in gel-vaccinated chicks. No differences were observed in mean antibody titres. Compared to spray, gel administration appears equally effective and less impactful on body temperature, thus supporting its application for IBV vaccination.

## 1. Introduction

Avian coronavirus, the causative agent of infectious bronchitis (IB), is one of the most relevant and impactful pathogens to affect the poultry industry worldwide [1]. IBV belongs to the genus *Gammacoronavirus*, family *Coronaviridae*, order *Nidovirales*, and is an enveloped virus with a single-stranded positive-sense RNA genome [2]. IBV is responsible for aspecific respiratory signs in chickens of every age, but which tend to be more severe at early ages. Depending on the pathogenicity of the strain involved, IBV may also damage the kidneys and oviduct, the latter resulting in drops in egg production and quality [3]. Mortality is usually low, but it may increase significantly in case of secondary infections by pathogens such as *E. coli* or *Mycoplasma* spp. Interactions with other respiratory or immunosuppressive viral diseases have also been documented [4].

The effective control of this highly contagious disease is essential for every type of production and is mainly achieved through rigorous biosecurity and mass vaccination [5]. Unfortunately, the efficacy of IBV vaccination is hindered by its remarkable evolutionary rate, ascribable to the frequent occurrence of both mutation and recombination events. This causes the emergence of a plethora of genetic IB variants, often found in cocirculation and coinfection and whose cross-protection is usually limited [6]. According to a phylogeny-based classification of the S1 gene sequence, a total of eight IBV genotypes are currently recognized, further divided into 36 lineages [7,8,9,10,11]. Based on which strains are circulating, several vaccination strategies can be adopted, either reliant on the administration of a homologous vaccine to the field strain or on a combination of heterologous vaccines able to provide a broader immunity, following the so-called “protectotype” concept [6,12].

In addition to the used vaccine strains, the conferred protection also depends on other factors related to vaccination procedures, including vaccine type, administration route and schedule [13]. Specifically, the broiler sector relies on the use of live attenuated vaccines, usually mass administered by drinking water or, more and more frequently, by spray [13]. The matter of vaccination timing is particularly debated. Multiple studies seem to suggest that the humoral immunity induced by early vaccination may be suboptimal, possibly because of interference with maternally derived antibodies (MDA), thus leaving the chicks unprotected against field viruses and favoring immuno-escape and recombination [14,15]. Despite this evidence, a clear trend towards hatchery administration has been observed recently in intensive poultry farming [16,17,18]. Moreover, even if an interval between subsequent administrations is traditionally recommended for a better immune response, likely to allow for tracheal epithelium recovery [19], the combined application of multiple vaccines at hatchery level has become the norm in many countries [16,17].

Hatchery vaccination by spray offers many advantages, such as lower labour costs and improved standardization of administration procedures and conditions compared to farm vaccination [6,17]. Several studies support its efficacy, with many vaccines being marketed as protective for the entire broiler cycle when administered at day-old [6]. On the other hand, it has been hypothesized that spray administration at the hatchery may result in a sudden drop in body temperature during a critical phase of chickens’ life, when the thermoregulatory system is not yet fully developed and they still act as poikilotherms [20], possibly affecting their growth and wellbeing [21].

Gel administration has recently drawn some interest as an alternative route for IBV vaccine delivery at the hatchery. Superabsorbent hydrogels are materials with a three-dimensional framework that retains stability even after absorbing large amounts of water. In the poultry sector, they have been used for a long time to supply live coccidiosis vaccines [22] and more recently probiotics and prebiotics [23]. Some studies have reported that IBV vaccination by gel allows for a proper immunization in experimental conditions [23,24,25,26]. Compared to spray vaccination, it may also have the additional advantage of a lesser impact on chicks’ body temperature [26].

The aim of this study was to assess the applicability of IBV gel vaccination at the hatchery in typical field conditions, by comparing gel administration with a newly developed patented system to spray vaccination, considered as the reference method.

## 2. Materials and Methods

### 2.1. Animals

The study was conducted on two groups of 50 chicks, one vaccinated by spray and the other by gel. The chicks were randomly selected from the same daily hatch of 114,300 Ross308 broilers, of which 109,300 were vaccinated by spray and 5000 by gel. Both groups were vaccinated at the hatchery with two live attenuated IBV vaccines, Cevac^®^ Ibird (strain 1/96, batch 2512H4D1KL) and Cevac^®^ Bron 120 L (strain H120, batch 1111H4D1KI) (Ceva Santé Animale, Libourne, France). Strain 1/96 belongs to the 793B lineage (GI-13), while strain H120 falls into the Mass lineage (GI-1). The combination of Mass and 793B vaccines was chosen as it is one of the most commonly adopted dual vaccination protocols [6,18], granting immunity against a broad range of variants [27,28].

### 2.2. Vaccination

The chicks were vaccinated in boxes of 90 birds each. Spray vaccination was administered using a SmartCount™ machine (Royal Pas Reform, Zeddam, The Netherlands), setting the droplet size at 200–250 µm (with ~10% of the volume of sprayed vaccine consisting of drops smaller than the average at 2.5 bar pressure). The spray dose per box was 15 mL (0.17 mL/chick), in which 90 doses of the two vaccines were mixed.

The newly developed SmartDrops (Phlatus ZRT, Perkáta, Hungary) application system, consisting of a drug-delivery superabsorbent hydrogel product and an application device, was used for gel administration. The implemented blue-colored hydrogel was based on superabsorbent polymers and aliphatic polycarboxylic acids, in accordance with European Union EC 1831/2003 standards. The application device allowed for an accurate delivery of the hydrogel by automatically controlling the shape, size, and size distribution of the droplets. Forty-five grams of gel, containing a mixture of 90 doses of the two vaccines, were administered to each box (0.5 g/chick).

### 2.3. Vaccine Intake

Vaccine intake was assessed by visually inspecting the chicks of both groups right after administration. Additionally, vaccine ingestion was evaluated in the gel-vaccinated group by individually checking whether the chicks’ tongue was blue-tinged after 15 min.

### 2.4. Body Temperature

The cloacal temperature of 25 randomly selected birds from each group was measured at take-off, and then 10 min and 1 h after vaccination. During that time, the two groups were kept in separate boxes in the chick holding room at 24 °C.

### 2.5. Bird Housing

After vaccination, the birds were individually identified with wing bands and the two groups were housed in separate pens. Every aspect of the management—housing, feed specification, lighting, etc.—was conducted following the Ross broiler manual recommendation [29].

### 2.6. Quantitative Real Time RT-PCR

Choanal swabs were collected from ten randomly selected chicks from each group at the first time-point at 4 days of age (DOA). The same animals were followed longitudinally by subsequent sampling at 8, 12, 16, 21, and 35 DOA. Spare samples were also taken from other individually identified birds in case of deaths among the initially selected chicks during the trial.

Each swab was individually eluted in 2 mL of PBS, and viral RNA was extracted with High Pure RNA Isolation Kit (Roche, Basel, Switzerland). Two previously validated real time quantitative RT-PCR (qRT-PCR) assays [30], one specific for Mass strains and the other for 1/96-like strains, were then used to assess vaccine coverage, titres and kinetics. The standard curves for the two assays were prepared by testing serial dilutions of the respective titrated vaccine, thus allowing an accurate quantification of vaccine titres. The implemented primers and probes are detailed in Table 1.

The assays were performed on LightCycler^®^ 96 Instrument (Roche Life Science, Penzberg, Germany) using SuperScript III Platinum One-Step qRT-PCR Kit (Invitrogen, Waltham, MA, USA). Titrated dilutions of Cevac^®^ IBird (1/96 strain) and Cevac^®^ Bron 120 L (H120 strain) vaccines were used as positive controls.

### 2.7. Serology

For serological monitoring, blood samples were taken from 16 animals of each group prior to vaccination to evaluate the presence of MDA, then at 21 and 35 DOA. The collected sera were tested at BioChek B.V. laboratories (Reeuwijk, The Netherlands) using CK119 IBV ELISA kit (Lot n.: FS7886).

### 2.8. Statistical Analyses

Differences among groups in terms of body temperature and antibody titres were analyzed with the two-sample *t*-test setting. Differences in viral titres were analyzed with the Kruskal–Wallis test, followed by the Mann–Whitney U test with Bonferroni adjustment as a post hoc test. For all analyses, the significance level was set at *p* < 0.05.

## 3. Results

### 3.1. Vaccine Intake

**Figure 1:**
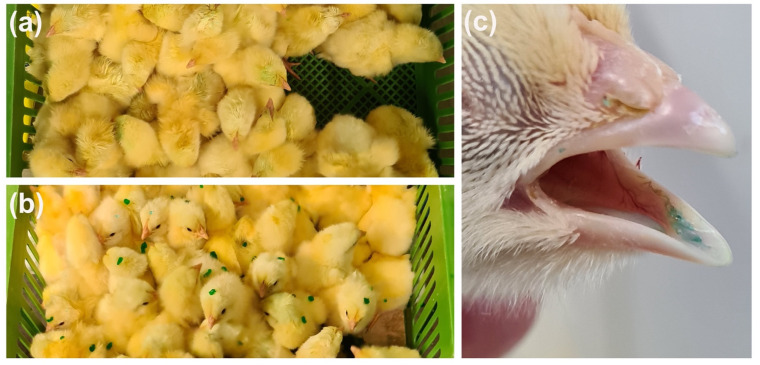
Post-administration evaluation of vaccine intake. Boxes of spray (**a**) and gel (**b**) vaccinated chicks immediately after vaccination, and tongue of a gel-vaccinated chick after 15 min from the administration (**c**).

After administration, the spray-vaccinated birds appeared wet and stained with the blue-dyed solution (Figure 1a), while gel droplets were visible on the group vaccinated by gel (Figure 1b). After 15 min, all gel-vaccinated chicks showed a blue-tinged tongue (Figure 1c).

### 3.2. Chick Temperature Following Vaccination

The body temperatures measured in the two groups are detailed in Table 2. A drop of more than 1 °C was observed in the spray-vaccinated group 10 min after administration, while the temperature of the gel-vaccinated birds remained stable. A slight decrease in temperature was observed in both groups after one hour in the chick holding room. The differences between the two groups were statistically significant at both sampling points.

### 3.3. Vaccine Coverage

**Figure 2:**
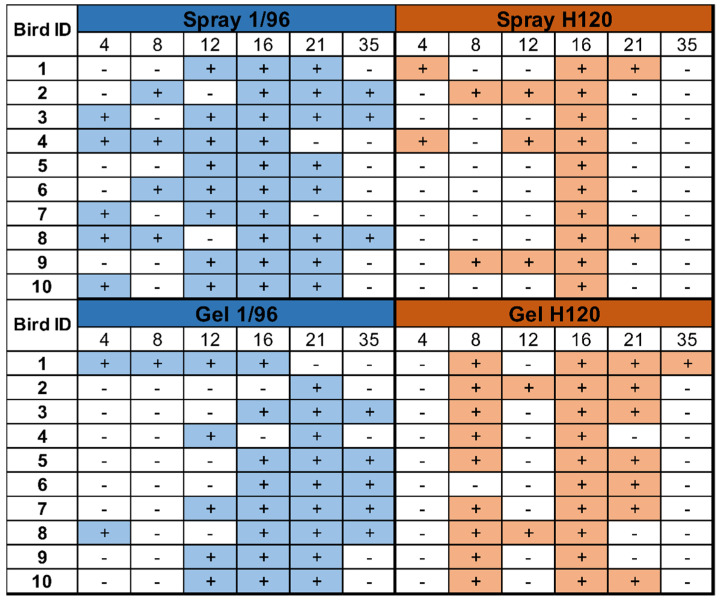
Individual qRT-PCR results. Individual samples from ten chicks per group and sampling point were evaluated with two qRT-PCR assays.

Detailed individual results from each group and sampling point are provided in Figure 2. All animals tested positive for H120 and 1/96 at least at one sampling point. For 1/96, some birds of both groups were already positive at day 4, and positive results were still observed at day 35. Individual 1/96 detections were more frequent in the spray-vaccinated group, especially in the first three time points. Conversely, a higher number of H120 detections were observed in the gel-vaccinated group, where the detection period (from 8 to 35 DOA) was slightly delayed compared to spray vaccination (from 4 to 21 DOA). In every group, the number of positive individuals peaked around day 16 and then started to decrease. Intermittent detections were also observed in both groups and for both vaccines.

**Figure 3:**
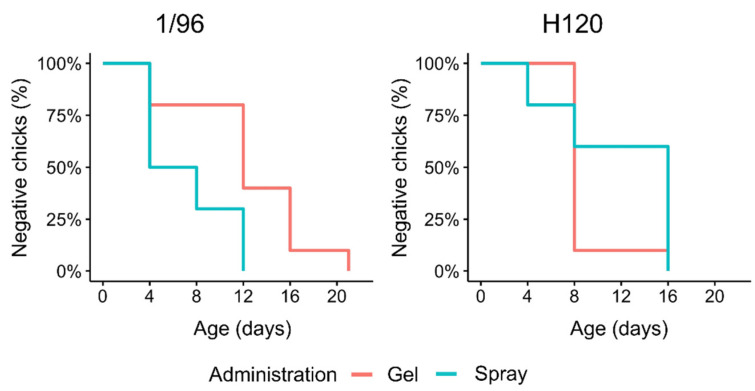
Percentage of negative chicks for vaccine strains 1/96 and H120. Kaplan–Meyer plots show the percentage of birds still negative for the two vaccines at the relative sampling point.

All animals of both groups were found positive for H120 by day 16. For 1/96, all animals were positive by day 12 in the spray-vaccinated group, and by day 21 in the gel-vaccinated one (Figure 3).

### 3.4. Vaccine Kinetics

**Figure 4:**
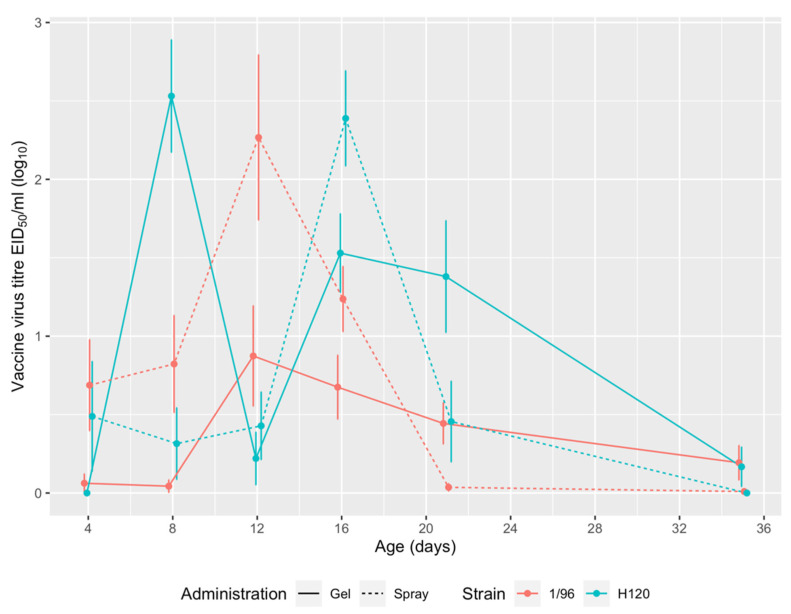
Replication of live attenuated IBV vaccines. For each group and sampling point, ten swabs were analyzed. Mean vaccine titres of positive results are expressed as log_10_ of embryo infectious doses 50 (EID_50_) per mL.

The replication of live attenuated IBV vaccines, administered by spray or gel, is detailed in Figure 4. 1/96 vaccine titres peaked at 12 DOA in both groups, showing a similar trend regardless of the route of administration. As for H120 vaccination, the observed titres peaked at day 8 in the gel-vaccinated group and at day 16 in spray-vaccinated animals. In both groups, H120 titres showed a temporary decline at 12 DOA, when 1/96 titres were at the highest point (Figure 4). No statistically significant differences between the two administration methods were detected at any of the sampling points, with the exception of H120 vaccination at 8 DOA.

### 3.5. Serology

**Figure 5:**
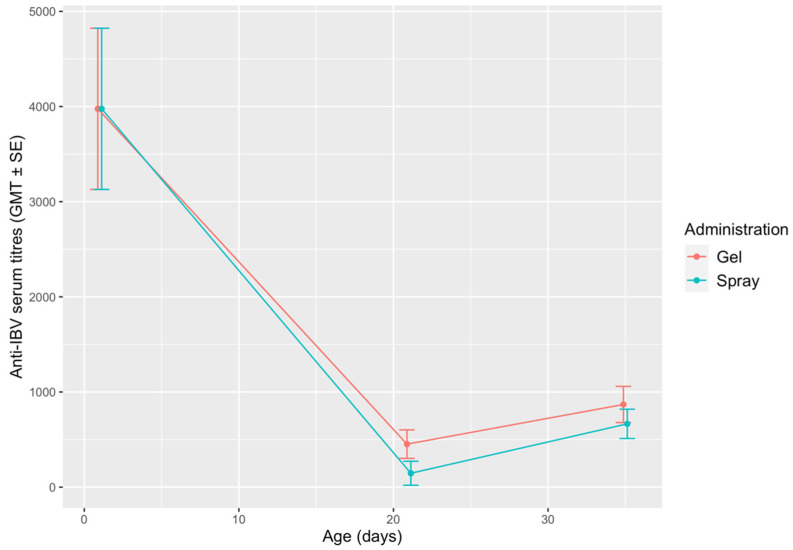
Mean anti-IBV antibody titres in the gel and spray-vaccinated groups. The results are shown as geometric mean titres (GMT) ± standard error (SE).

Anti-IBV antibody titres detected in the two groups are shown in Figure 5. Regardless of the route of administration, the titres observed in 21-day-old-chicks were significantly lower than the MDA titres observed at the hatchery, while a slight rise was observed at 35 DOA. The differences between the two groups were not statistically significant at any sampling point.

## 4. Discussion

Based on the herein presented comparison, the administration of multiple IBV live vaccines at the hatchery yielded similar results when performed by spray, which is the routinely adopted method, and by gel. In typical field conditions, the two routes of administration were comparable in terms of coverage and elicited immune response, as shown by vaccine replication (Figure 4) and antibody titres (Figure 5). For 1/96 strain, a 100% coverage was reached earlier (12 DOA) in the gel-vaccinated birds than in the spray group (20 DOA). For both vaccine strains, intermittent detections were observed, in agreement with previous observations by Tucciarone et al. [26]. This finding could be tentatively ascribed to the persistent circulation of the live vaccine virus among chicks of the same group, which may elicit subsequent reinfections.

No statistically significant differences were found in serology. MDA titres were high before vaccine administration in both groups, which belonged to the same hatch. A marked decline was observed at 21 DOA, followed by a slight increase at 35 DOA. This trend is consistent with the results of previous works [15,27,31]. The low antibody levels observed at 21 and 35 DOA may be a consequence of MDA interference, particularly since vaccines were administered at the hatchery [15]. However, this should not be interpreted as a vaccination failure, as it is well-established that humoral antibody titres correlate poorly to the protection conferred by IBV vaccines [6,27].

On the other hand, protection seems to be associated to cellular and local immunity at the tracheal level [27]. Despite not allowing a direct evaluation of the elicited immune response, the assessment of vaccine viral titres at this level, that are thought to compete with field strains for tracheal receptors [12,30], is commonly used as a proxy for vaccination quality and efficacy [6,30].

Slight differences were observed between the two groups in terms of vaccine kinetics. Vaccine strain H120 was detected from 4 to 21 DOA in the spray-vaccinated group, and from 8 to 35 DOA in the gel-vaccinated one, with comparable titres between the two groups. Strain 1/96 was found at every sampling point (from 4 to 35 DOA) in both groups, with slightly higher titres in the spray-vaccinated birds until 16 DOA and in the gel-vaccinated ones at later sampling points. However, the observed differences may be deemed inconclusive to decide whether the two supply methods differ in terms of vaccine take, and therefore in potential efficacy.

The comparison of the vaccine kinetics observed in the gel vaccinated group with those observed in an experimental setting [30] reveals a substantially overlapping pattern. Although absolute titres were slightly different, this could be ascribed to the differences in study conditions and/or implemented Mass vaccine (based on strain B48 instead of H120).

No notable signs were observed in the two groups for the duration of the trial, and the growth performances were in line with Broiler Ross standards (data not shown). Even if a rigorous assessment of vaccination efficacy and safety was beyond the scope of this work, this may be seen as proof supporting the safety of gel vaccination, at least comparatively to spray. While the two administration methods provided similar results in terms of coverage, gel vaccination did not affect the chicks’ body temperature, contrary to spray vaccination which caused a 1 °C drop in body temperature within an hour after administration. A decrease equal to roughly 2 °C was previously observed in the hatchery where the study was conducted, when 20 mL of spray were administered to each box (data not shown). Cold stress at the hatchery is reported to affect muscle growth and development [32] and could even predispose to necrotic enteritis [33]. However, it is worth noting that the observed drop in temperature, even if statistically significant, may not be enough to cause any problem [34], and further studies are needed to prove the biological significance of this side effect of spray vaccination.

Besides the actually implemented Mass and 793B vaccines, whose combination represents one of the most commonly adopted IBV vaccination protocols [27,28], gel administration is likely suitable for every other live IBV vaccine with similar features. Likewise, further research efforts may be devoted to assessing whether gel delivery systems are suitable for other vaccines that are commonly administered at the hatchery against different diseases. Gel administration also allows to effectively combine different substances, such as coccidiosis vaccines and probiotics, granting their stability [23]. Being able to administer IBV vaccines with other active principles would be of great practical value, allowing for a holistic approach to chicks’ health and early immunization while minimizing stressful procedures during a critical phase of the cycle.

Complementing the first studies conducted in experimental conditions, the present results provide further evidence supporting the feasibility of IBV hatchery vaccination by gel in field conditions. Considering its possible benefits over traditionally implemented methods, it would be worth testing this technique in standardized in vivo challenge experiments involving different vaccines and field strains, to better characterize its features and provide definite evidence on its efficacy.

## Figures and Tables

**Table 1:** Primers and probes implemented in the Mass and 1/96-like specific real time RT-PCR assays, previously designed by Tucciarone et al. [30].

Primer/Probe	Sequence
1/96-like Forward primer	5′-CCTGGTTCAGGTTGGCATTT-3′
1/96-like Reverse primer	5′-ATGCACTGCCTGCATTGTTG-3′
1/96-like Probe	5′-FAM-TCTACTGCATAAGCACCCCCATG-BHQ1-3′
Mass Forward primer	5′-AGCAGACGCAGGTTTGGCTA-3′
Mass Reverse primer	5′-TGGTTGACATCTTCGCAAGG-3′
Mass Probe	5′-FAM-CATCTGGTTCCATAGACATCTTTGTCG-BHQ1-3′

**Table 2:** Average body temperatures (°C) and standard deviations measured in the spray and gel-vaccinated groups.

	Take-Off	Spray-Vaccinated Chicks (*N* = 25)		Gel-Vaccinated Chicks (*N* = 25)	
		After 10 min	After 1 h	After 10 min	After 1 h
Average	40.9	39.6	39.2	40.8	40.2
SD	0.19	0.70	0.39	0.27	0.24

## Data Availability

Not applicable.
